# A phase II randomised (calibrated design) study on the activity of the single-agent trabectedin in metastatic or locally relapsed uterine leiomyosarcoma

**DOI:** 10.1038/s41416-018-0190-y

**Published:** 2018-07-30

**Authors:** Angiolo Gadducci, Federica Grosso, Giovanni Scambia, Francesco Raspagliesi, Nicoletta Colombo, Giovanni Grignani, Paolo Casali, Roberta Sanfilippo, Angela Buonadonna, Armando Santoro, Milena Bruzzone, Grazia Artioli, Domenica Lorusso, Elena Biagioli, Roldano Fossati, Francesca Galli, Emanuele Negri, Eliana Rulli, Valter Torri, Maurizio D’Incalci

**Affiliations:** 1 Azienda Ospedaliero-Universitaria Pisana, Pisa, Italy; 2 Azienda Ospedaliera Nazionale SS. Antonio e Biagio e Cesare Arrigo, Alessandria, Italy; 3 Fondazione Policlinico Universitario A. Gemelli IRCCS Università Cattolica, Rome, Italy; 4 Fondazione IRCCS Istituto Nazionale dei Tumori, Milan, Italy; 5 Istituto Europeo di Oncologia, Milan, Italy; 6 Università di Milano-Bicocca, Milan, Italy; 7 Candiolo Cancer Institute FPO IRCCS, Candiolo, Turin Italy; 8 Università degli Studi di Milano, Milan, Italy; 9 Centro di Riferimento Oncologico, Pordenone, Aviano Italy; 10 Istituto Clinico Humanitas, Milan, Rozzano Italy; 11 Ospedale Policlinico San Martino, Genova, Italy; 12 Azienda Ulss 3 Serenissima, Venice, Italy; 13 IRCCS – Mario Negri Institute for Pharmacological Research, Milan, Italy

**Keywords:** Cancer, Drug development

## Abstract

**Background:**

Patients with recurrent/metastatic uterine leiomyosarcoma (U-LMS) have a dismal prognosis. This phase II study aims to evaluate trabectedin efficacy and safety in advanced U-LMS.

**Methods:**

Eligible patients had received ≥ one line of chemotherapy. Gemcitabine ± docetaxel naive patients were randomised to Arm A: trabectedin 1.3 mg/m^2^ or calibration Arm B: gemcitabine 900 mg/m^2^ and docetaxel 75 mg/m^2^. Patients who had already received gemcitabine ± docetaxel directly entered Arm A. Primary end-point: 6-month progression-free rate (PFS-6). The null hypothesis that the true PFS-6 = 14% was tested against a one-sided alternative. This design yielded a 5% type I error rate and 90% power when the true PFS-6 is 25%.

**Results:**

Overall, 126 patients entered Arm A (45 from randomisation and 81 directly) and 42 Arm B. Arm A patients characteristics: median age = 57; ≥2 previous chemotherapy lines = 37.4%; metastatic disease = 93%. The study met the condition for trabectedin activity: PFS-6 = 35.2% (95% CI: 26.2–45). No difference in PFS by the number of previous chemotherapy lines emerged. Median OS = 20.6 months (IQR: 8–36.4). In Arm B, the PFS-6 = 51.5% (95% CI: 33.5–69.2). No toxic deaths occurred. In Arm A, only 4 patients interrupted treatment for toxicity.

**Conclusions:**

Trabectedin is active and well tolerated, retaining similar efficacy across one to three previous lines of chemotherapy.

## Introduction

Uterine leiomyosarcoma [U-LMS] accounts for 1.3% of all uterine malignancies, with an estimated annual incidence of 0.55 per 100,000 women.^1^ Literature data report survival rates of ~50% for early-stage disease,^2–5^ but U-LMS has a great tendency to local and distant recurrence. Although distant relapses involve lungs and upper abdomen, the metastatic potential is very wide and distant lesions can be found everywhere.^6^

Patients with metastatic disease at diagnosis, or with early recurrence after initial treatment, except for a subset of patients with completely resectable disease, have a dismal prognosis and usually their median survival is <1 year. Chemotherapy is the standard treatment in this clinical setting, wherein there are no curative therapeutic options with the noteworthy exception of surgery for metastases isolated to the lung.^7–13^ The medical treatment is similar to that used for adult-type soft-tissue sarcoma [STS]s and includes anthracyclines, ifosfamide and dacarbazine both as single agent and in combination regimens.

Single-agent gemcitabine in a phase II Gynecologic Oncology Group trial obtained an objective response [OR] rate of 20.5% among 42 patients with recurrent or persistent U-LMS, most of whom had received prior chemotherapy or radiotherapy.^14^

In the last decade, the combination of fixed-dose rate infusion of gemcitabine + docetaxel has emerged as a promising option both as first- and second-line treatment of locally unresectable or metastatic U-LMS.^15,16^

The addition of the human antiplatelet-derived growth factor receptor-α monoclonal antibody olaratumab to doxorubicin achieved a significant improvement of 11.8 months in median overall survival (OS) in a recent phase Ib/II trial including 133 patients with locally advanced or metastatic STSs not previously treated with anthracyclines.^17^ Both the Food and Drug Administration [FDA] and European Medicines Agency (EMA) have granted accelerated approval of olaratumab, combined with doxorubicin, as first-line therapy for doxorubicin-naive patients with inoperable STS.

Trabectedin is a marine-derived agent, that has obtained marketing authorisation from EMA for the treatment of advanced STSs after failure of anthracyclines and ifosfamide.^18^ Trabectedin forms adducts in the minor groove of DNA, and triggers a cascade of events that interfere with several transcription factors, DNA binding proteins, and DNA repair pathways, resulting in G2-M phase cell cycle arrest and apoptosis.^19^ Trabectedin also modifies tumour microenvironment, particularly by reducing the number of Tumour Associate Macrophages (TAM) and the production of inflammatory cytokines and chemokines^20–22^ responsible for enhancing angiogenesis, tumour growth and downregulating antitumour immunity.^23^ It is worth noting that macrophage infiltration and CSF1 response signature have been reported to be predictors of poor prognosis of leiomyosarcoma patients,^24^ thus suggesting that the ability of trabectedin to target TAM might be therapeutically relevant in this disease. These anti-inflammatory and immunomodulatory properties may have a major, possibly synergistic, role in the antitumour activity of trabectedin.^25^ Steroid pre-medication significantly reduced hepatotoxicity and myelosuppression, which are the most frequent side effects of the drug.^26^ Sensitivity to trabectedin is increased in cells with deficient homologous recombination repair.^27,28^

Distinct sarcoma histotypes are recognised to be sensitive to specific cytotoxic drugs in the metastatic setting^29,30^ and trabectedin has shown very promising activity in highly pretreated U-LMS.^31–35^

The present study specifically aimed at evaluating the activity of trabectedin as second/further line of treatment in persistent, recurrent or metastatic U-LMS pretreated with chemotherapy.

## Patients and methods

### Study design and patients

This is a multicentre, randomised, non-comparative phase II study. Eligible patients satisfied these inclusion criteria: histologically proven persistent, recurrent or metastatic U-LMS; ≥1 previous systemic treatment (either adjuvant or first-line metastatic setting) with anthracycline ± ifosfamide or gemcitabine ± docetaxel; measurable disease, as defined by Response Evaluation Criteria in Solid Tumors, version 1.1 (RECIST 1.1 criteria); ECOG Performance Status ≤ 2; age ≥ 18 years; ≥3weeks since prior antitumour therapy; recovery from toxic effects of prior therapies to National Cancer Institution Common Toxicity Criteria [NCI-CTC] grade ≤ 1; adequate haematological, renal, and liver function. Exclusion criteria: prior exposure to trabectedin; peripheral neuropathy grade ≥ 2; history of other malignancies; known central nervous system metastases; serious concomitant illnesses.

Patients not previously treated with gemcitabine were randomised to single-agent trabectedin (experimental arm) or the combination of gemcitabine + docetaxel, whereas those previously treated with gemcitabine were directly included in the experimental arm. Patients randomised to gemcitabine + docetaxel served as calibration arm whose aim was to point out possible biases by checking the similarity of the results obtained in this group with the historical controls. This indirect comparison is intended to improve the reliability of experimental arm results. At progression, patients randomised to the calibration arm could be crossed to and included in the trabectedin arm.

### Treatment plan

Patients enrolled into experimental arm received trabectedin at a dose of 1.3 mg/m^2^, via a central venous catheter as a 24-h infusion on day 1 of 21-day treatment cycle. When this study was planned the dosage of trabectedin in U-LMS across different clinical series ranged between 1.0 and 1.5 mg/m^2^ daily. The schedule of 1.3 mg/m^2^ 24-h continuous infusion used in this study was considered to be the best balance between efficacy and toxicity according to the limited evidences then available.^31,32,35^ Pre-medication for trabectedin was 8 mg oral dexamethasone the day before receiving trabectedin and 12 mg iv dexamethasone on day 1 of each treatment cycle, 30 min prior trabectedin. Treatment was administered until progressive disease, major toxicity, patient’s intolerance or unwillingness to continue treatment, or at physician’s discretion.

Patients enrolled into the calibration arm received gemcitabine: 900 mg/m^2^ iv on days 1 and 8 over 90 min, followed by docetaxel: 75 mg/m^2^ on day 8 iv over 1 h. Treatment was administered every 3 weeks for six cycles. After six cycles, responding patients could receive two additional cycles of therapy or continue with gemcitabine alone until progression, unacceptable toxicity, patient’s intolerance or unwillingness to continue treatment, or medical decision by the responsible physician. Recommended pre-medication for docetaxel was oral dexamethasone: 8 mg twice a day starting the day prior to the infusion and continuing for three days. Prophylactic granulocyte-colony stimulating factor: 150 μg/m^2^ on days 9 and 15, or peg-filgrastim: 6 mg on day 9 or 10 was given in patients receiving gemcitabine + docetaxel.

All adverse events (AEs) were assessed at each cycle and were graded according to the NCI-CTC, version 3.0.

### Response evaluation and follow-up procedures

A physical examination and a radiological examination were performed before the start of treatment, at week 8, 16 and 24 from enrolment, then every 3 months, until disease progression or death. RECIST 1.1 criteria were used for disease assessments.

### Statistical analysis

The primary study end-point was the progression-free survival rate at 6 months [PFS-6], defined as the rate of patients alive and progression-free at 6 months from study entry. The PFS-6 was assessed in the per protocol (PP) population that included all patients without major violations of eligibility criteria who had received at least two cycles of treatment. Subjects who had not progressed or died before 6 months and without a disease evaluation in the period between the 22nd and the 27th week were not categorised as progression-free and were not considered evaluable for the primary analysis, unless the absence of disease progression was confirmed in the disease evaluations after the 27th week. The primary end-point was provided with its 95% confidence interval [95% CI].

According to empirical evidences gathered from the analyses of phase II trials in pretreated patients, trabectedin was considered insufficiently active with a PFS-6 equal or below 14%^36^ and sufficiently active with a PFS-6 equal or above 25%. Using an A’Hern single-stage design for phase II trials^37^ and assuming that PFS-6 for trabectedin given as second or further line would be similar, 109 patients were needed to be enrolled in the trabectedin arm to reject the null hypothesis of activity < 14% with a power of 90% and a one-sided type I error of 5%. Trabectedin was considered sufficiently active if at least 22 patients were alive and progression-free at 6 months.

Secondary end-points of the study were PFS, OS, and the toxicity profile. The secondary efficacy end-points were evaluated in the PP population. PFS was defined as the time between the study entry and the progression or death for any cause. Subjects who have not recurred or died while on study were censored at the last disease assessment date. OS was defined as the time between the study entry and death, regardless of the cause of death. Subjects who were not reported as having died at the time of the analysis were censored at the date they were last known to be alive. Survival curves were estimated by using the Kaplan–Meier [KM] method and compared with the log-rank test. The toxicity profile was evaluated in the safety population that included all patients without major violations of the eligibility criteria who had received at least one treatment dose. Adverse events were graded according to NCI-CTC version 3.0. For any single toxicity, the incidence of events and the maximum grade experienced by each subject were provided. Continuous variables were expressed as medians with inter-quartile ranges. All analyses were performed using SAS software, versions 9.4 (SAS Institute) and a two-sided *p*-value < 0.05 was considered significant.

## Results

**Fig. 1:**
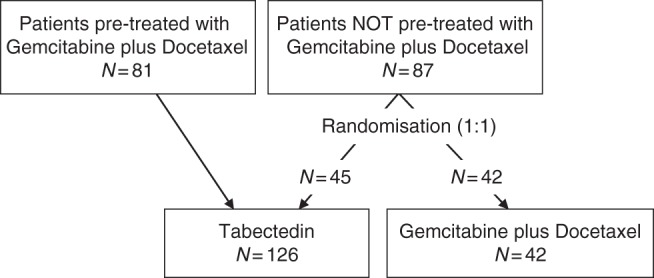
Flow diagram for patients with partially platinum-sensitive ovarian cancer who were accrued into the trial

From April 2010 through January 2016, 168 women with persistent/recurrent or metastatic U-LMS already treated with chemotherapy were entered into this trial from 26 Italian Centres. The trial’s diagram flow is shown in Fig. 1.

### Experimental arm—Trabectedin

**Fig. 2:**
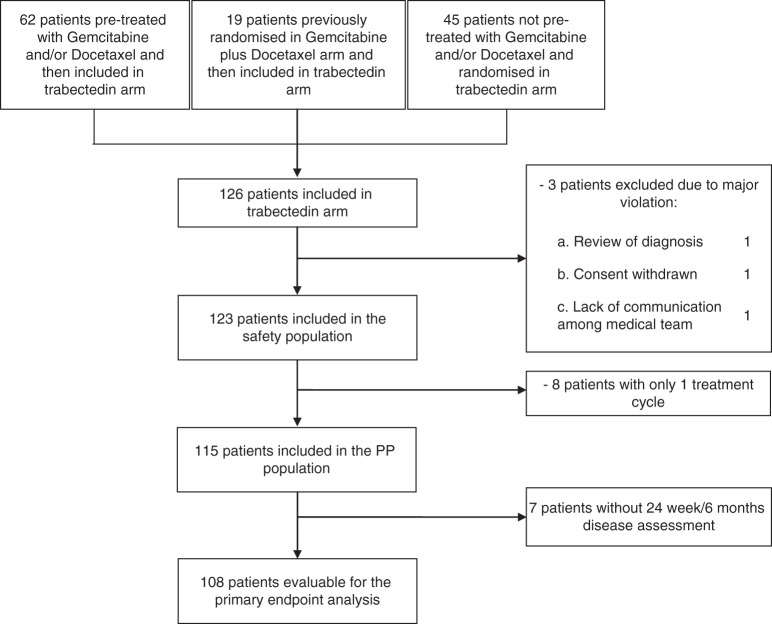
CONSORT trial flow diagram for patients with partially platinum-sensitive ovarian cancer who were accrued into the Trabectedin Arm

Overall, 126 patients were included and Fig. 2 shows the patients flow-chart and defines the populations for safety, primary and secondary end-points analyses.

Table 1 shows the clinical characteristics of the patients included in the PP population.

**Table 1:** Tumour characteristics at first diagnosis and prior treatments—PP population

	Trabectedin *N* = 115
Stage at first diagnosis, *n* (%)	
IA	9 (8.0)
IB	49 (43.4)
IIA	10 (8.8)
IIB	4 (3.5)
IIIA	8 (7.1)
IIIB	2 (1.8)
IVA	9 (8.0)
IVB	22 (19.5)
Missing	2
Surgery for primary disease, *n* (%)	115 (100)
Total abdominal hysterectomy + bilateral salpingo oophorectomy	88 (76.5)
Hysterectomy	25 (21.7)
Other	2 (1.7)
Lymphadenectomy, *n* (%)	22 (22.4)
Missing	17
Adjuvant radiotherapy, *n* (%)	14 (12.2)
Site of external beam radiotherapy, *n* (%)	
Pelvic	12 (92.3)
Other	1 (7.7)
Missing	1
Previous chemotherapies, *n* (%)	
Only adjuvant	40 (34.8)
Only first-line	32 (27.8)
Adjuvant plus first-line	23 (20.0)
First and second-line	15 (13.0)
Adjuvant plus first-line and second-line	4 (3.5)
Adjuvant plus first-line (unknown second-line)	1 (0.9)
Adjuvant chemotherapy, *n* (%)	68 (59.1)
Anthracyclines	41 (60.3)
Gemcitabine	27 (39.7)
First-line chemotherapy, *n* (%)	75 (65.2)
Anthracyclines	39 (52.0)
Gemcitabine	34 (45.3)
Other	2 (2.7)
Second-line chemotherapy, *n* (%)	19 (16.7)
Anthracyclines	2 (10.5)
Gemcitabine	12 (63.2)
Other	5 (26.3)

Median age of patients was 57 (range: 34–76) and 54 (range: 33–74) years at study entry and at first diagnosis, respectively.

Notably, 72 (62.6%) patients had received only one line of chemotherapy, whereas the remaining 43 (37.4%) patients had undergone two or three previous chemotherapy lines. As shown in Table 2, only 8 (7%) patients had disease confined to the pelvis while in the great majority (93%) the disease had already spread to distant sites, mostly to the lung (65.2%).

**Table 2:** Tumour characteristics at baseline—PP population

	Trabectedin *N* = 115
Status of disease at study entry, *n* (%)	
Progression	79 (68.7)
Recurrence	33 (28.7)
Persistent	3 (2.6)
Site of disease at study entry, *n* (%)	
Only pelvic	8 (7.0)
Only distant metastasis	71 (61.7)
1 site of metastasis	35 (49.3)
2 sites of metastasis	22 (31.0)
3 sites of metastasis	9 (12.7)
>3 sites of metastasis	5 (7.0)
Pelvic plus distant metastasis	36 (31.3)
1 site of metastasis	16 (44.4)
2 sites of metastasis	11 (30.6)
3 sites of metastasis	6 (16.7)
>3 sites of metastasis	3 (8.3)
Peritoneum, *n* (%)	39 (33.9)
Liver, *n* (%)	18 (15.7)
Spleen, *n* (%)	4 (3.5)
Lung, *n* (%)	75 (65.2)
Bone, *n* (%)	18 (15.7)
Intra-abdominal lymph nodes, *n* (%)	10 (8.7)
Extra-abdominal lymph nodes, *n* (%)	9 (7.8)
Abdominal wall, *n* (%)	5 (4.3)
Other, *n* (%)	19 (16.5)

Adherence to treatment was satisfactory as trabectedin was discontinued due to causes independent of disease progression in just 24 (19.5%) cases and, of these, only 4 (16.7%) for toxicity. A median of 3 cycles of trabectedin (inter-quartile range [IQR]: 2–6; range: 1–59) was administered in the patients who interrupted the treatment because of disease progression. When disease progression was not the cause of treatment interruption, the median number of trabectedin cycles was 11.5 (IQR: 6-13; range: 1–41). In the PP population, a complete (CR) or partial response (PR) was observed in 8 (7.0%) and 19 (16.5%) patients, respectively. Forty-three (37.4%) patients reached a stable disease (SD) and 45 (39.1%) progressed. Out of 108 patients evaluable for primary analysis, 69 (63.9%) progressed, one (0.9%) died and 38 were alive and progression-free after 6 months from the study entry, therefore the PFS-6, i.e., the primary end-point of this study, was 35.2% (95% CI: 26.2–45). After a median follow-up of 34 months, 102 (88.7%) patients progressed or died. Overall, 34 (27.6%) patients were able to receive 10 or more cycles of trabectedin.

**Fig. 3:**
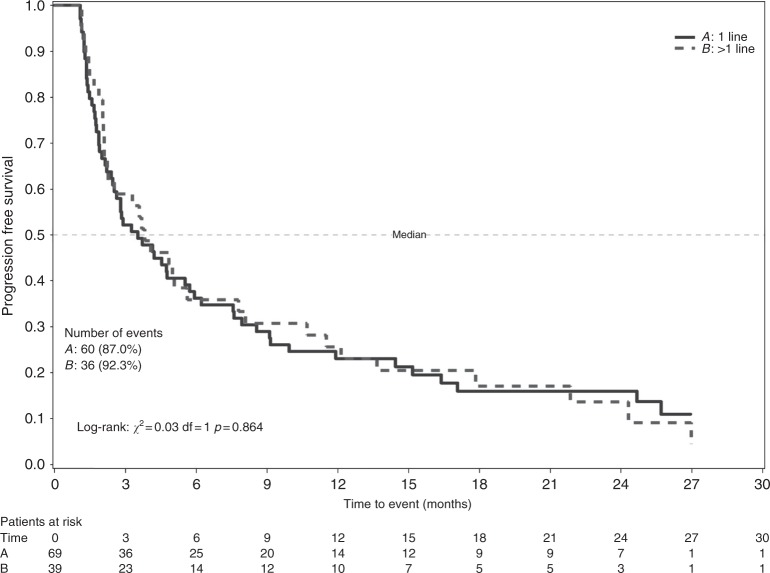
Kaplan–Meier display of progression-free survival according to previous chemotherapy lines—PP population, Trabectedin Arm

Median PFS was 4.1 months (IQR: 1.9–10.7). Suppelentary Figure 1A shows the KM curves of PFS. No difference in PFS according to the number of previous chemotherapy lines was detected (log-rank test *p* = 0.864), as shown in Fig. 3.

During the study period, 76 (66.1%) patients died. Median OS was 20.6 months (IQR: 8–36.4). Supplemntary Figure 1B shows the KM curves of OS.

The trabectedin safety analysis population included 123 patients. No treatment-related deaths occurred. Overall, there were 1430 adverse events [AEs], 923 (64.5%) grade 1, 352 (24.6%) grade 2, 130 (9.1%) grade 3, and 25 (1.7%) grade 4. The incidence of patients with at least one grade 3 or 4 AE was 48%. Table 3 summarises the frequencies and grade of specific AEs. Seventeen serious AEs [SAEs] (of which 5 had a probable or definitive causal relationship with treatment) occurred in 16 patients (12.7%).

**Table 3:** Maximum toxicities

Toxicity	G1 or G2 *n* (%)	G3 *n* (%)	G4 *n* (%)	G3 + G4 *n* (%)
Trabectedin safety population (*N* = 123)				
Allergic reaction	2 (1.6)	1 (0.8)	0 (0.0)	1 (0.8)
Haemoglobin	37 (30.1)	5 (4.1)	1 (0.8)	6 (4.9)
Leucocytes/WBC	33 (26.8)	12 (9.8)	3 (2.4)	15 (12.2)
Neutrophils	25 (20.3)	17 (13.8)	10 (8.1)	27 (22.0)
Platelets	6 (4.9)	3 (2.4)	2 (1.6)	5 (4.1)
Atrial fibrillation	1 (0.8)	1 (0.8)	0 (0.0)	1 (0.8)
Fatigue	32 (26.0)	4 (3.3)	0 (0.0)	4 (3.3)
Infusion site extravasation	0 (0.0)	1 (0.8)	0 (0.0)	1 (0.8)
Hyperglycaemia	1 (0.8)	1 (0.8)	0 (0.0)	1 (0.8)
Anorexia	14 (11.4)	1 (0.8)	0 (0.0)	1 (0.8)
Constipation	30 (24.4)	2 (1.6)	0 (0.0)	2 (1.6)
Diarrhoea	5 (4.1)	1 (0.8)	0 (0.0)	1 (0.8)
Nausea/vomiting	46 (37.4)	1 (0.8)	0 (0.0)	1 (0.8)
Obstruction, colon	0 (0.0)	0 (0.0)	1 (0.8)	1 (0.8)
Perforation, bowel	0 (0.0)	1 (0.8)	0 (0.0)	1 (0.8)
Alkaline ph. increased	4 (3.3)	1 (0.8)	0 (0.0)	1 (0.8)
ALT increased	19 (15.4)	7 (5.7)	0 (0.0)	7 (5.7)
AST increased	17 (13.8)	6 (4.9)	1 (0.8)	7 (5.7)
Cholecystitis	0 (0.0)	1 (0.8)	0 (0.0)	1 (0.8)
GGT increase	8 (6.5)	4 (3.3)	0 (0.0)	4 (3.3)
Hepatotoxicity	7 (5.7)	4 (3.3)	0 (0.0)	4 (3.3)
Infection/fever	13 (10.6)	2 (1.6)	0 (0.0)	2 (1.6)
C.P.K. increase	5 (4.1)	2 (1.6)	0 (0.0)	2 (1.6)
Hyponatraemia	0 (0.0)	1 (0.8)	0 (0.0)	1 (0.8)
Pain	18 (14.6)	1 (0.8)	1 (0.8)	2 (1.6)
Dyspnoea	7 (5.7)	1 (0.8)	0 (0.0)	1 (0.8)
Pulmonary embolism	0 (0.0)	1 (0.8)	0 (0.0)	1 (0.8)
Creatinine	6 (4.9)	0 (0.0)	1 (0.8)	1 (0.8)
Thrombosis, vein	0 (0.0)	1 (0.8)	1 (0.8)	2 (1.6)
Gemcitabine + docetaxel safety population (*N* = 39)				
Haemoglobin	25 (64.1)	6 (15.4)	0 (0.0)	6 (15.4)
Leucocytes/WBC	10 (25.6)	8 (20.5)	2 (5.1)	10 (25.6)
Neutrophils	7 (17.9)	9 (23.1)	5 (12.8)	14 (35.9)
Platelets	13 (33.3)	6 (15.4)	1 (2.6)	7 (17.9)
Fatigue	11 (28.2)	1 (2.6)	0 (0.0)	1 (2.6)
Dermatitis	1 (2.6)	1 (2.6)	0 (0.0)	1 (2.6)
Enteritis	0 (0.0)	0 (0.0)	1 (2.6)	1 (2.6)
Perforation, rectum	0 (0.0)	0 (0.0)	1 (2.6)	1 (2.6)
Infection/fever	9 (23.1)	1 (2.6)	1 (2.6)	2 (5.1)
Oedema, limb	0 (0.0)	1 (2.6)	0 (0.0)	1 (2.6)
Neuropathy, sensory	3 (7.7)	1 (2.6)	0 (0.0)	1 (2.6)
Pain, abdomen	2 (5.1)	1 (2.6)	0 (0.0)	1 (2.6)
Fistula, genitourinary	0 (0.0)	1 (2.6)	0 (0.0)	1 (2.6)
Hydronephrosis	0 (0.0)	1 (2.6)	0 (0.0)	1 (2.6)

### Calibration arm—gemcitabine + docetaxel

Overall, 42 patients were randomised in the calibration arm. Three patients were excluded from all the analysis populations due to protocol major violations. All patients started the treatment, but one patient underwent only one cycle of chemotherapy, therefore 39 and 38 patients were included in the safety and in the PP population, respectively. Finally, 5 patients were excluded from primary end-point analysis because they had the 6-month tumour assessment outside the correct time frame, as described in the statistical analysis paragraph.

Median age of patients was 55 (range: 33–72) and 52 (range: 32–72) years at study entry and at first diagnosis, respectively. As for the trabectedin group, the disease had already spread to distant sites in about the 90% of patients, with lung as the most frequent localisation (52.6%). Further details of tumour characteristics at first diagnosis and baseline are shown in supplementary Tables 1 and 2. In patients who interrupted the treatment because of disease progression a median number of 3 doses of gemcitabine + docetaxel (IQR: 2.5–6; range: 2–13) was administered. When disease progression was not the cause of treatment interruption, the median number of cycles in the calibration arm was 7 (IQR: 6–8; range: 1–16).

In the PP population, a CR or PR was observed in 5 (13.2%) and 6 (15.8%) patients, respectively. Fifteen (39.5%) patients reached a SD and 12 (31.6%) progressed. Out of 33 patients evaluable for primary analysis, 16 (48.5%) progressed, no one died and 17 were alive and progression-free after 6 months from the study entry, therefore the PFS-6 was 51.5% (95% CI: 33.5–69.2). After a median follow-up of 29 months, 34 (89.5%) patients had progressed or died and 16 (42.1%) patients died. Median PFS was 6.9 months (IQR: 2.4–15.4) and median OS was 36.7 months (first quartile equal to 13.7, third quartile not reached).

Thirty-nine patients treated with gemcitabine + docetaxel were included in the safety population. No treatment-related deaths occurred. Overall, there were 557 AEs, 302 (54.2%) grade 1, 179 (32.1%) grade 2, 63 (11.3%) grade 3, and 13 (2.3%) grade 4. The incidence of patients with at least one grade 3 or 4 AE was 61.5%. Table 3 summarises the frequency and grade of specific AEs. Ten SAEs (of which 2 had a probable causal relationship with treatment) occurred in 10 patients (23.8%).

## Discussion

In the present investigation, the PFS-6 for patients treated with trabectedin was 35.2% (95% CI: 26.2–45) and such result is consistent with the literature. A pooled analysis combining 5 phase II studies, overall including 62 patients with U-LMS previously treated with a median of two lines of chemotherapy, reported that trabectedin obtained a PFS-6 = 30.7%.^35^ A retrospective analysis assessed 66 patients pretreated with a median number of 3 chemotherapy lines who received trabectedin at two European sarcoma reference centres in a 10-year time interval.^33^ The PFS-6 was 33%, and the median OS was 14.4 months. The post-hoc subset analysis of a phase 3 trial assessed 232 patients with advanced U-LMS after failure of anthracycline-based chemotherapy.^34^ Trabectedin arm showed a greater clinical benefit rate and a longer median PFS when compared with a dacarbazine arm (31% versus 18%, *p* = 0.051 and 4 versus 1.5 months, *p* = 0.0012, respectively). In this trial, trabectedin was given at a higher dose (1.5 mg/m^2^, 24-h intravenous infusion) but efficacy appeared similar while grade 3 and 4 haematologic toxicities and transaminases increase were more frequent than in our study.

It is noteworthy that in our study, the activity of trabectedin seems to be independent of the number of prior chemotherapy lines and that 25 and 10% of patients treated with trabectedin received at least 10 and 14 cycles, respectively, irrespective of the numbers of previous chemotherapy lines. This reflects trabectedin favourable toxicity profile and prolonged tumour control in a significant proportion of patients.

The toxicity profile of trabectedin was as expected, with low rate of G3-G4 haematological and non-haematological toxicities. Steroid pre-medication had probably a crucial role in the prevention of major toxicities.^26^ Severe transaminases increase was observed in 5.7% of patients but was reversible and not cumulative and not associated with signs or symptoms.

Trabectedin can be combined with other agents with manageable toxicity. In a phase II trial, first-line treatment with trabectedin (1.1 mg/m^2^/3 h infusion) and doxorubicin (60 mg/m^2^) induced G3-4 neutropaenia, thrombocytopaenia, anaemia, increased transaminases and fatigue in 78%, 37%, 27%, 39% and 19%, respectively, of 108 patients with advanced STS or U-LMS.^38^ Of the 47 patients with U-LMS, 28 reached a PR and 13 reached SD with an overall clinical benefit of 87.2%.

The same combination of trabectedin and doxorubicin did not show superiority over single-agent doxorubicin (75 mg/m^2^) as first-line treatment in a phase II randomised study including 115 patients with advanced STSs.^39^

Regarding the gemcitabine + docetaxel arm, the performance of this calibration arm was as expected, being in line with the results reported by Hensley et al in their series of 48 patients in second-line therapy for metastatic U-LMS (PFS rate at 24 weeks: 52%, 95% CI: 37.2–66.7%).^16^ To ameliorate the toxicity profile, the docetaxel dose recommended in the TAUL study was 75 mg/m^2^, instead of 100 mg/m^2^ used by Hensley et al.^15,16^ Indeed, G3-G4 leukopaenia, thrombocytopaenia, anaemia and pulmonary toxicity occurred in 25.6%, 17.9%, 15.4% and 0% of patients in our series, and in 22.9%, 39.6%, 25% and 8.3% of the patients treated in the Hensley’s study. The clinical benefit of the addition of docetaxel to single-agent gemcitabine has been debated for long. At the time of this study planning the results of the French randomised TAXOGEM study were not yet available.^40^ The TAXOGEM study showed that, in the 42 evaluable patients with U-LMS, median PFS in patients treated with gemcitabine alone and with gemcitabine + docetaxel was similar: 5.5 and 4.7 months, respectively, and single-agent gemcitabine was associated with less toxicities. In the TAUL study, the gemcitabine + docetaxel arm served only for calibration and not as a control group. The general assumption about a calibration experimental design is that whenever the data do not support the hypothesis that the expected activity prevails in the calibration group, the investigational group results might be declared suspect and a second trial recommended.^41^ Indeed, both experimental and calibration results fulfilled expectations, ensuing reliability to the clinical evidence that trabectedin is to be considered active and well tolerated in pretreated patients with recurrent/metastatic U-LMS, achieving a PFS-6 of 35% with a substantial proportion of patients with long-term control of disease.

Trabectedin, as monotherapy, is a valid therapeutic option in relapsing patients even after a combination treatment such as gemcitabine + docetaxel. Extended pathological characterisation and next generation sequencing transcriptomic studies are strongly warranted to identify biomarkers associated with prolonged clinical benefit.

### Data availability

Data supporting the results reported in the article can be found at IRCCS Mario Negri Institute for Pharmacologic Research, Milan. Data sharing is encouraged and data are available on request to the corresponding author

## Electronic supplementary material

Supplementary figures and tables

## Availability of data and material

Data supporting the results reported in the article can be found at IRCCS Mario Negri Institute for Pharmacologic Research, Milan. Data sharing is encouraged and data are available on request to the corresponding author.
