# Supplementary material for: A phase II randomised (calibrated design) study on the activity of the single-agent trabectedin in metastatic or locally relapsed uterine leiomyosarcoma
Source: Br J Cancer. 2018 Jul 30;119(5):565–71. doi: 10.1038/s41416-018-0190-y (PMC6162262; doi:10.1038/s41416-018-0190-y)
Supplement: Supplementary file 1 — Supplementary figures and tables [file 41416_2018_190_MOESM1_ESM.docx]

Figure 1 Supplemental, Panel A.

Kaplan-Meier display of progression free survival - PP population, Trabectedin Arm


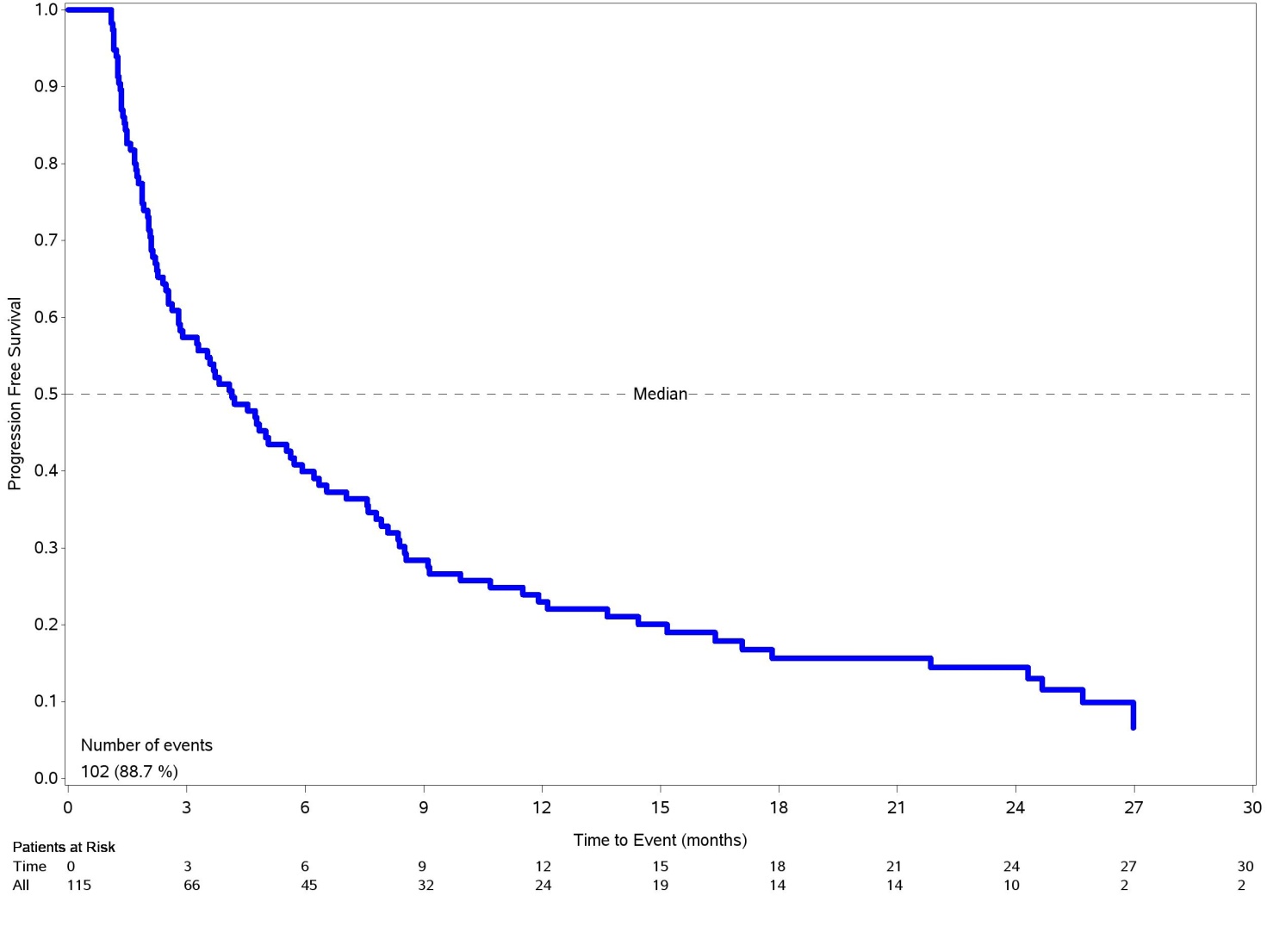


Figure 1 Supplemental, Panel B.

Kaplan-Meier display of overall survival - PP population, Trabectedin Arm


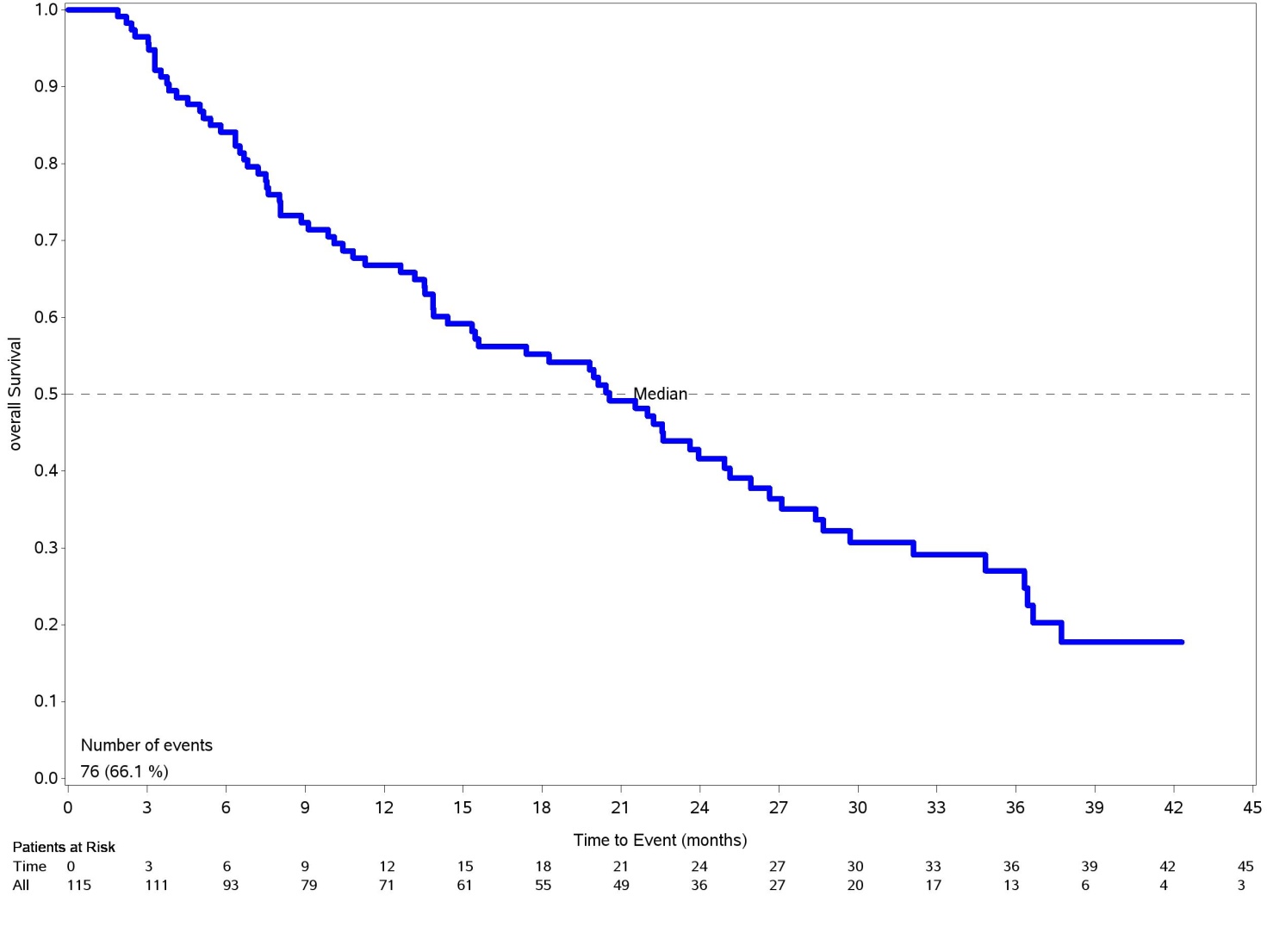


Supplementary Table 1 Tumor characteristics at first diagnosis and prior treatments - PP population

|  | **Gemcitabine+Docetaxel**  **N=38** |
| --- | --- |
| **Stage at first diagnosis - n (%)** |  |
| IA | 7 (18.4) |
| IB | 14 (36.8) |
| IIA | 2 (5.3) |
| IIB | 4 (10.5) |
| IIIA | 2 (5.3) |
| IIIB | 1 (2.6) |
| IVA | 4 (10.5) |
| IVB | 4 (10.5) |
| **Surgery for primary disease - n (%)** | 38 (100) |
| Total abdominal hysterectomy+  bilateral salpingo oophorectomy | 29 (76.3) |
| Hysterectomy | 9 (23.7) |
| **Lymphadenectomy - n (%)** | 11 (28.9) |
| **Adjuvant radiotherapy - n (%)** | 8 (21.1) |
| **Site of external beam radiotherapy - n (%)** |  |
| Pelvic | 8 (100) |
| **Chemotherapies - n (%)** |  |
| Only adjuvant | 24 (63.2) |
| Only first line | 13 (34.2) |
| First and second line | 1 (2.6) |
| **Adjuvant chemotherapy - n (%)** | 24 (63.2) |
| Anthracyclines | 24 (100) |
| **First line chemotherapy - n (%)** | 14 (36.8) |
| Anthracyclines | 14 (100) |
| **Second line chemotherapy - n (%)** | 1 (2.6) |
| Anthracyclines | 1 (100) |

Supplementary Table 2 Tumor characteristics at baseline - PP population

|  | **Gemcitabine+Docetaxel**  **N=38** |
| --- | --- |
| **Status of disease at study entry - n (%)** |  |
| Progression | 21 (55.3) |
| Recurrence | 17 (44.7) |
| Persistent | 0 (0.0) |
| **Site of disease at study entry - n (%)** |  |
| Only pelvic | 4 (10.5) |
| Only distant metastasis | 18 (47.4) |
| 1 site of metastasis | 12 (66.7) |
| 2 sites of metastasis | 4 (22.2) |
| 3 sites of metastasis | 1 (5.6) |
| >3 sites of metastasis | 1 (5.6) |
| Pelvic plus distant metastasis | 16 (42.1) |
| 1 site of metastasis | 8 (50.0) |
| 2 sites of metastasis | 3 (18.8) |
| 3 sites of metastasis | 3 (18.8) |
| >3 sites of metastasis | 2 (12.5) |
| **Peritoneum - n (%)** | 15 (39.5) |
| **Liver - n (%)** | 7 (18.4) |
| **Spleen - n (%)** | 1 (2.6) |
| **Lung - n (%)** | 20 (52.6) |
| **Bone - n (%)** | 2 (5.3) |
| **Intra-Abdominal Lymph nodes - n (%)** | 5 (13.2) |
| **Extra-Abdominal Lymph nodes - n (%)** | 2 (5.3) |
| **Abdominal wall - n (%)** | 4 (10.5) |
| **Other- n (%)** | 3 (7.9) |
